# miRNAs as Biomarkers in Diabetes: Moving towards Precision Medicine

**DOI:** 10.3390/ijms232112843

**Published:** 2022-10-25

**Authors:** Maria Alexandra Angelescu, Octavian Andronic, Simona Olimpia Dima, Irinel Popescu, Irit Meivar-Levy, Sarah Ferber, Daniela Lixandru

**Affiliations:** 1 Faculty of Medicine, University of Medicine and Pharmacy “Carol Davila”, 050474 Bucharest, Romania; 2 University Emergency Hospital, 050098 Bucharest, Romania; 3 Center of Excelence in Translational Medicine (CEMT), Fundeni Clinical Institute, 022328 Bucharest, Romania; 4 Academy Nicolae Cajal Institute of Medical Scientific Research, Titu Maiorescu University, 040441 Bucharest, Romania; 5 Orgenesis Ltd., Ness Ziona 7414002, Israel; 6 Department of Human Genetics, Sackler School of Medicine, Tel Aviv University, Tel Aviv 6997801, Israel; 7 Department of Biochemistry, University of Medicine and Pharmacy “Carol Davila”, 050474 Bucharest, Romania

**Keywords:** microRNA, biomarkers, insulin resistance, precision medicine

## Abstract

Diabetes mellitus (DM) is a complex metabolic disease with many specifically related complications. Early diagnosis of this disease could prevent the progression to overt disease and its related complications. There are several limitations to using existing biomarkers, and between 24% and 62% of people with diabetes remain undiagnosed and untreated, suggesting a large gap in current diagnostic practices. Early detection of the percentage of insulin-producing cells preceding loss of function would allow for effective therapeutic interventions that could delay or slow down the onset of diabetes. MicroRNAs (miRNAs) could be used for early diagnosis, as well as for following the progression and the severity of the disease, due to the fact of their pancreatic specific expression and stability in various body fluids. Thus, many studies have focused on the identification and validation of such groups or “signatures of miRNAs” that may prove useful in diagnosing or treating patients. Here, we summarize the findings on miRNAs as biomarkers in diabetes and those associated with direct cellular reprogramming strategies, as well as the relevance of miRNAs that act as a bidirectional switch for cell therapy of damaged pancreatic tissue and the studies that have measured and tracked miRNAs as biomarkers in insulin resistance are addressed.

## 1. Introduction

Type 1 diabetes mellitus (T1DM) is a complex metabolic disease with increasing incidence over the past decade, being classified as an autoimmune disease that destroys β-pancreatic islets, leading to a dysfunction in insulin production and subsequent hyperglycemia, which is the main cause of acute and chronic complications in diabetes [1].

Several environmental and genetic factors have been described, including but not limited to infections occurring during the early stages of life having certain mutations, the most frequent being HLA (human leucocyte antigen) class II or autoantibodies that target the insulin-secreting β cells of the pancreas [1]. Studies have also shown that the pathogenic path leading to T1DM consists of a deregulation of Foxp3+ Treg cells (regulatory T cells) and an autoimmune driven infiltration β-cells of Langerhans islets with autoreactive T cells [2,3].

While much is yet unknown, studies have found that noncoding RNA (ribonucleic acid), such as miRNA (microribonucleic acid), can play an important role [1]. miRNAs are single-stranded, endogenous small noncoding RNAs (18–25 nucleotides). The utility of using miRNAs as biomarkers resides in their presence in bodily fluids, thus making them relatively easy to detect. They are also stable and have a particular circuit, as they are taken up by protein matter such as exosomes into different tissues in order to fulfil their role in transcription. Specific miRNAs could therefore be of great clinical use, not only in diagnostics, but also in predicting complications [4]. Exosomal miRNAs are under investigation as potential disease biomarkers, as well as important factors in islet transplantation due to their role in donor–host communication as valuable links between immune cells. A better understanding of the noncoding RNA might lead to more advantageous long-term post-transplantation results [5].

Type 2 diabetes mellitus, however, is a manifestation of IR (insulin resistance), a defective response to insulin and a subsequent altered glucose metabolism. Strongly linked with other metabolic factors, such as obesity, metabolic syndrome and altered lipid homeostasis, insulin resistance is the product of several parts of a well-regulated pathological pathway. The aforementioned pathway goes all the way from the insulin receptor to the GLUT 4 transporter (glucose transporter type 4) which regulates glucose uptake. Each step is correlated with several miRs, some of which have an important role in pathogenesis and could have therapeutical uses [6].

This review aimed to evaluate miRNAs as biomarkers in diabetes with the potential to stage the progression of different modes of signaling pathways that regulate cellular direct reprogramming strategies as therapeutic tools in diabetes treatment. We also address studies that measured and tracked miRNAs as biomarkers in insulin resistance.

## 2. The Biochemical Substrate of β-Cell Destruction and miRNAs’ Roles in Its Modulation

Understanding the biosynthesis of non-coding RNA and its role in gene regulation is essential. The aforementioned process consists of several steps that take place firstly in the nucleus and afterwards in the cytoplasm. An enzyme called RNA polymerase II is given the task of transcribing, and therefore, pri-miRNA is sliced in order to form pre-miRNA and, finally, into mature miRNA [2]. The multitude and variety of miRNA sequences is of great importance in maintaining proper homeostasis and functionality. Thus, malfunctions that occur in processes in which miRNAs play a key factor can lead to immunity disruption. There is a strong correlation between miRNAs, Treg cells and the presence of autoantibodies. The latter have proved to appear prior to the clinical onset of diabetes. Although much is still unknown regarding the processes that lead to pathogenesis, extensive research has been conducted concerning the role of T regulation cells in such autoimmune diseases as T1DM [7].

miRNA142-3p has been found to have an important role in regulating Tregs through Tet2 (methyl cytosine dioxygenase). Another biochemical compound strongly correlated with Treg cell function is miRNA-91a. Among its role in dysregulating Treg cells, thus leading to the possible pathogenesis of diabetes, it also has an effect on modulating T follicular helper cell precursors which is strongly correlated with diabetes onset. Meanwhile, high miRNA-181a values could indicate an upregulation of T cell activation. This goes to show T-cells’ and miRNAs roles in β-cell autoimmunity pathogenesis [2]. In addition to T regulatory cells, another biochemical compound that miRNA has a strong correlation to is CXCL 10 (C-X-C motif chemokine ligand 10) [8]. Due to the fact of its many roles in immunity regulation, CXCL 10 has recently been taken into consideration and studied carefully. It was previously observed to have high tissular or serum values in patients suffering from T1DM and, thus, was reviewed in its interrelation with miR-16-5p. The results show that the latter decreased while the former increased. Further research is needed in order to prove what kind of effect miR-16-5p might have through CXCL10 pathways [8].

## 3. miRNA as an Early Marker of Islet Autoimmunity

miRNAs have been found to have diverse roles within the endocrine pancreas (see Table 1), which makes them key components of islet autoimmunity in T1DM. 

One of the key factors in regulating T1DM is preserving residual insulin secretion for as long as possible, as it could lead to better therapeutic outcomes and fewer complications. This is currently quite difficult as a diagnosis for T1DM often comes late, when peptide C is already decreasing. The importance of new and effective biomarkers could ease this problem and aid in a quicker pre-symptomatic diagnosis [8]. Circulating RNA can be detected through PCR (polymerase chain reaction) and gene sequencing in the peripheral blood of patients who exhibit T1DM [9]. One of the most promising miRNAs whose function has been explored so far is miRNA-375 due to its role in insulin expression, which has been observed in vitro, and its high level of expression within β cells. Therefore, considering data previously extracted from KO (knockout) diabetic mice, serum levels have been monitored. This has been observed in afebrile children. miRNA-375 was dosed at an early onset as subcutaneous insulin had not yet been administered. The children had already tested positive for autoantibodies which would suggest a β cell injury [9,10]. The miRNA-375 levels were not corelated with any other biomarkers for DM. However, when studying its function in human islets exposed to high levels of glucose, its important role was confirmed. While miRNA-375 is downregulated in contexts of high exposure to glucose, it is upregulated in controlled T1DM [10]. Thus, a link between glucotoxicity and miRNA-375 could exist. However, extracellular miRNA-375 is not specific to β cells and could have other tissular origins. It has also been shown to have a role in viral infections, which could correlate with a possible viral cause for T1DM. Low values have also been found to correlate with loss of β cells, subsequent proliferation of alfa cells and a decrease in kidney function. The latter has been observed by the urinary presence of miR-21 in individuals who also expressed a high risk for severe kidney failure [10,11].

miRNA-409-3p was also analyzed in NOD diabetic mice and people diagnosed with T1DM. The results showed that decreased values correlated with the presence of diabetes mellitus. The pathways through which this phenomenon occurs were immunity-related and did not merely pertain to glucose levels. The immune cells that mostly strongly correlated to mi-RNA-409-3p were peripheral T cells. However, these associations were not replicated in patients who had been living with DM over a long period, thus suggesting the biomarker’s sensitivity to acute and severe insulitis. This theory is further supported by the higher expression of miRNA-409-3p during the administration of aCD3 in diabetic mice [12].

Furthermore, miR-409-3p has proved to be non-specific to T1DM and also found to have significance in other autoimmune diseases, such as rheumatic arthritis. Until its cellular origin can be precisely located, its applicability is still debatable, although a combined array of other RNA fragments could be looked into [12].

miRNA-155 has been observed in several autoimmune diseases due to the fact of its interrelations with white blood cells, specifically in the activation processes, and because of this, it has also been observed in children with T1DM by comparing levels with healthy counterparts. The results showed an increase in serum levels. This could also be possibly correlated with ZnT8 (zinc transporter 8) antibodies [13].

The aforementioned observations also noted a concomitant decrease in miRNA-326 and miRNA-146a. miRNA-146a found values could be correlated with the increase of proinflammatory cytokines, such as IL-6 (interleukin-6) and α-interferon, due to their observed association [14].

miRNA-204 has been found to be released from dying β cells in the early stages of the disease. This occurs because of the presence of inflammatory markers in the autoimmune process of pancreatic β cell destruction, with levels being directly proportional to the amount of cell apoptosis. Elevated levels have been found in at-risk individuals who presented DM specific antibodies, but also in recently diagnosed patients as levels seem to lower over time along with disease progression and complete loss of insulin secretion [15].

Contrary to other miRNAs, miRNA-204 has been proven to be specific to T1DM as it has not been detected at abnormal values in T2DM (type 2 diabetes mellitus) or other autoimmune diseases. It is also present in adult onset T1DM, which could have great clinical applicability for differential diagnosis with T2DM [15]. Furthermore, the family of miRs-211-5p and -204-5p has been found to be an important steppingstone in regulating apoptotic factors in β-cell islets. Their inhibitory role consists of modulating pro-apoptotic transcription proteins, resulting in activation of caspase-3 [15]. Similarly, extracellular vesicles containing miRNA 21-5p are also under investigation as a possible indicator of β cell damage. Studies have found an increase after administration of inflammatory cytokines such as IL-1β, INT-γ (interferon-γ) and TNF-α (tumor necrosis factor α), which is suggestive of a proinflammatory characteristic. The chosen cytokines were meant to mimic the inflammatory pattern found in T1DM. While some findings have shown a decrease in circulating EV (extracellular vesicles), such as miRNA 21-5p in children with recent onset, others have shown an increase in adults with long withstanding T1DM. We must also consider the disease’s heterogenic characteristics, as well as the multi-tissular origin of mi-RNAs [16]. A cluster of three miRNAs, noted as miR-23~27~24, have been shown to be upregulated in children with major peptide C loss. This indicates it to be an appropriate test for predicting β cell apoptosis [17]. Another study observed miR-1225-5p and miR-320 in relation to early β cell damage in NOD (non-obese diabetic) and STZ (streptozotocin-induced) mice, taking into account the necessary adjustments for age and BMI (Body Mass Index) as these variables could also have an impact upon miRNA circulatory levels. Thus, weight loss in obesity could dysregulate the already modified circulatory miRNAs. These findings suggest that the aforementioned miRNAs could not only detect early islet destruction but also monitor progression of the disease. Further research with a larger sample size is necessary in order to confirm these findings [18].

miRNA-103a-3p was first noticed in T2DM and afterward shown to have upregulated levels in type 1 Brazilian patients. Obese diabetic mice have shown improvement in glucose levels after silencing of this miRNA. A significant effect upon miR107 has also been observed. As it has an effect upon caveolin-1, this gene was seen to also be upregulated in vivo. miRNA levels were also abnormal in the livers of patients who presented with associated comorbidities to T2DM [19,20].

Several upregulated miRNAs have also been found in T1DM lactating mothers by observing levels in comparison with healthy mothers. One such example is hsa-miR-26a-5p. This compound has been linked to HBA1c (hemoglobin A_1c_), which is important in diabetes mellitus diagnosis. Further associations with β-cell failure and the pathophysiology of T1DM onset have also been observed [21].

A separate entity on its own, MODY3 (model onset diabetes of the young) has also been taken into consideration while scanning for potential miRNAs due to the fact of its heterogenic genetic component. Due to the fact of its being frequently misdiagnosed as type 1 diabetes, a quicker method other than genetic testing would be useful for a differential diagnosis. The disease is often correlated with a deficiency in HNF1A (hepatocyte nuclear factor 1 homeobox A) protein which has been studied along with several mi-RNAs. miRNA-200-3p and miR378a-5p have been indicated as worthy of further research. Difficulties could arise due to the very small percentage of diabetes cases attributed to MODY3 [22].

HNF1A is an essential transcription factor which has been strongly associated with the regulation of SGLT2 co-transporter and several markers of acute inflammation, such as CRP (C-reactive protein). Furthermore, along with several other members of the HNF gene family, it makes up an important pancreatic, hepatic and renal regulatory glucose framework. Thus, mutations in HNF1A, most correlated with MODY pathogenesis, have been found to dysregulate β-cell production and insulin regulation. Identified mutations have been found to range from deletions to insertions to missense or nonsense mutations and even whole-gene deletion. Different sites where the mutations may take place can have different consequences. For example, if mutations are localized in the promoter region this will affect other transcription factors that target hepatic and pancreatic tissue. On the other hand, mutations within the dimerization domain will end up affecting protein synthesis [23].

HNF1A is known to be part of a chain of positive regulation within embryogenesis, thus being an important steppingstone in iPSs differentiation into fully functioning insulin-producing pancreatic cells. Its interactions within the HNF1A-HNF4 loop further regulates PDX1 [24].

Research has shown HNF1A-AS1 (also known as HASTER) to be responsible for regulating HNF1A levels. Therefore, a higher expression results in downregulation of HNF1A through feedback mechanisms and vice versa. This has been proved by observing HNF1A functions in HNF1A-AS1-deficient mice. This dual modulation provides an important stabilizing function.

Several lncRNAs have also been found to have regulatory roles on haploin sufficient genes, among which HNF1A is mentioned. These fine regulatory mechanisms are important in diabetes pathogenesis and put forth noncoding DNA as an essential pillar that needs to be better studied [25].

Among other miRNAs whose correlation with β-cell dysfunction has been implied, miR-132-3p, miR-101-3p, miR-148b-3p and miR-1275 are mentioned in the literature [9,19].

**Table 1:** Well-known miRNAs and their role in metabolism regulation of pancreatic development and insulin biosynthesis, secretion and signaling.

	miRNAs for Pancreatic β-Cell Function		References
1	miR-21	Overexpression of miR-21 in MIN β-cells decreased *GSIS* and was associated with a decrease in VAMP2, a SNARE protein essential for β-cells exocytosis and GTPase (guanosine triphosphatase)	[26,27,28]
2	miR-24 miR-26	miR-24 down-regulated NeuroD1 and Hnf1-α Deletion of Dicer1 in adult β-cells decreased by 70% of insulin mRNA levels that was associated with increased levels of transcriptional repressors Sox6 and Bhlhe22 [29] In β-cells Dicer 1-deficient mice decreased the levels of 4 miRNAs (i.e., miR-24, miR-26, miR-182 and miR-148) which led to diabetes due to the significant decreases in insulin mRNA [30] Overexpression of miR-24 in MIN6 β-cell decreased both cell proliferation and GSIS via the ROS/PKC (reactive oxygen species/protein kinase C) pathway and resulted in pancreatic β-cell dysfunction	[29,30,31,32]
3	miR-29	Three isoforms of miR-29 were all highly expressed in primary mouse islets [33] and also in the serum of T2DM [34] involved in insulin secretion and β-cell apoptosis In prediabetes, negatively regulated GSIS, and miRNA-29a was up-regulated in β-cells by glucose and decreased GSIS played a role in cytokine-induced β-cell dysfunction Increased miR-29 in response to exposure to proinflammatory cytokines, and also promoted apoptosis by diminishing levels of the antiapoptotic protein Mcl1 miRNA-29b-3p inhibited GSIS by targeting the SNARE protein	[33,34,35,36,37]
4	miR-199b-5p	Targeted the mRNA of miR-199b-5p involved in β-cell proliferation Up-regulation of miR-199b-5p and miR-375 additively promoted the proliferation of β-cells	[38]
5	miR-30	Highly expressed in human fetal pancreas miR-30d was associated with increased expression of Mafa and consequently promoted insulin gene transcription in pancreatic β-cells miR30d directly targeted TNF-α-induced MAP4K4 and prevented the inhibitory effect of MAP4K4 on the expression of Mafa and IRS2, leading to the partial recovery of TNF-α-induced suppression on insulin production and insulin secretion miR-30a-5p played a key role in glucotoxicity and targeted NeuroD1	[39,40]
6	miR-17-3p	Involved in the proliferation/survival of pancreatic β-cells	[41]
7	miR-320d miR-320c miR-320a	Inhibited insulin resistance through IRS-1 regulating ERK1/2 inhibited its phosphorylation signaling pathway (in patients with polycystic ovary syndrome) miR320a inhibited the Wnt/β-catenin signaling pathway by targeting the 3′UTR of β-catenin mRNA β-catenin was a direct target gene of miRNA-320a in liver cancer cells (negative regulator, which in turn, regulated the Wnt/signaling pathway) miR320a was inversely associated with HCC proliferation in HVV cell lines (i.e., HepG2, BEL-7402 and SMMC-7721) miR-320a and miR320c initiated expression at the beginning of liver differentiation (endoderm differentiation) via *HNF-6*	[42,43,44,45,46]
8	let-7b-5p let-7f-5p let-7d-5p let-7i-5p let-7a-5p let-7c-5p let-7g-5p let-7e-5p	Targeted IRS2 and regulated β-cell insulin signaling One of the regulators of matrix metalloproteinase (MMP) expression and function let-7 acted as a negative regulator of hepatic differentiation in human adipose tissue-derived stem cells (hADSCs) by deletion of the HNF-4α transcription factor	[47,48,49]
9	miR-22-3p	Elevated hepatic miR-22-3p expression impaired gluconeogenesis by silencing the Wnt-responsive transcription factor Tcf7 Critical modulator of the Wnt signaling pathway	[50]
10	miR-15a miR15b miR-16miR-195	Panel in pancreas regeneration (“New Pancreas from Old: Microregulators of Pancreas Regeneration”) vs. known to demonstrate a limited potential to proliferate in adult life Targeted Ngn3 (a downstream target of HNF6) after partial pancreatectomy (but not during embryonic development) Overexpression of miR-16-5p reduced the total number of endocrine cells	[51,52]
11	miR-19a-3p	Targeted NeuroD1 (negative regulation) and decreased insulin mRNA miR-19b overexpression in MIN6 cells, and it was found that the endogenous NeuroD1 protein was reduced, while inhibition of the endogenous miR-19b had little effect on the expression of NeuroD1 miR-19b downregulated insulin 1 gene expression through targeting of NeuroD1, and by doing so regulated the differentiation and functioning of β-cells	[53]
12	miR-124a	Has been shown to regulate Foxa2 (a TF crucial for early pancreas development) Decreased level of Foxa2 protein, and the major downstream target genes of Foxa2 decreased, including PDX-1, Kir6.2 and sulphonylurea receptor 1 (key protein for normal development and function of the pancreas) miR-124a and miR-23a were expressed early during pancreas organogenesis	[54,55,56]
13	miR-7	miRNA-7 inhibited tumor metastasis and reverses EMT through AKT/ERK1/2 inactivation by targeting EGFR in epithelial ovarian cancer Overexpression of miR-7 suppressed the capacities of cell invasion and migration and, in turn, morphological changes from a mesenchymal to an epithelial-phenotype occur	[57,58]
14	miR-200	β-cell specification; maintenance of the epithelial phenotype Family members: miR-200a, miR-200b, miR-200c, miR-141 and miR-429 Highly abundant in β-cells and functions to repress glucagon expression by targeting the transcription factors cMaf and Fog2 (both stimulate glucagon gene expression) miR200a was induced during the differentiation of hPSCc (human pluripotent stem cells) to insulin-producing β-islet like cells by targeting ZEB1 and ZEB2 involved in EMT by repressing E-cadherin expression.	[59,60]
15	miR-375	Pancreas development Insulin biosynthesis Insulin secretion	[61,62,63]

## 4. The Role of miRNA Detection during Pancreatic Islets Transplantation as a Potential Marker for Damaged Pancreatic Tissue

Islet transplantation is a procedure considered for treating T1DM which has been uncontrollable through insulin administration. However, through immune mechanisms, many grafts are rejected by the organism, making it quite unsustainable. Recent studies show that if we could carefully monitor pancreatic function post-transplantation, we will be able to develop new action plans to ensure better functioning of islet grafts. Rather than use classical tests, such as peptide C and HbA_1c_ levels, exosomes have been observed as a possible useful biomarker [5]. Exosomes are vesicles containing several types of nucleic acids, along with other important molecules that are then transported beyond cellular walls through exocytosis. Upon further analysis of their function, it has been noticed that miRNAs thus isolated can point to several pathological processes happening within the pancreatic islets. Under several stressor conditions, different types of miRNAs will be detected, along with other types of small RNAs [5]. Exosomes could also have further applicability in T1DM, as they do not pose a significant risk of ulterior toxicity and would be easy to administer. This conclusion was reached by studying the possibility of auto-transplantation in vitro and in mouse islets. Exosomes derived from stem cells have been thus shown to have multiple effects. A protection from apoptosis by regulating genes such as Bad, Bax and Bcl-2 and a surge in insulin mRNA value have been noticed. However, insulin-resistant mice did not respond well as the islet inflammation advanced [5]. Mesenchymal stem cells are not the only source from which exosomes can be derived, with an observed improvement over glycemic function. Those derived from bone marrow and menstrual cells could improve immune function, through T-cell populations or by stimulating insulin production, as shown in murine models [64].

### 4.1. Cell-Replacement Therapy for Diabetes

To avoid grafts rejected by the immune response we also approached data on the trans-differentiation (TD) process in relation to different organs to the pancreas for generating functional insulin-producing cells from adult human cells. The appeal of using TD in contrast to stem cell-derived β cells would be the practicality of working with acinar cells or non-β pancreatic cells. In fact, similarities between the different cells of the endocrine pancreas could be of great use, as the literature suggests that they could all differentiate into β cells upon extensive apoptosis of the β cells. These cells represent the most advantageous targets for regeneration due to the fact of their epigenetic similarities to β cells and the possibility of their undertaking insulin synthesis [65,66]. Furthermore, a particular stage in which β cells are found, which is of relevance considering our research, consists of immature β cells. These are present in endocrine islets and have been shown in mice to have the capacity to differentiate into β cells [66]. Ductal cells could also provide a viable option for the generation of new β cells through epithelial to mesenchymal transition (EMT). This has been shown to take place in a hypoglycemic state, along with EGF (epidermal growth factor), although some authors have attributed it to inflammatory cytokines, rather than low glycemic levels. Similarly, using ductal epithelial cells from the gallbladder has been looked into, as it is a non-essential organ and the issue of donors would be resolved. These findings have yet to be sufficiently developed for clinical use [66]. Both the EMT and the mesenchymal to epithelial transition (MET) have been observed as processes that are undergone during organogenesis. These stages, along with the existent intermediate stages that have been observed in embryonic cells, open up various possibilities of cell-replacement therapy for diseases such as DM [67].

While EMT represents a conversion from an apical cell polarity, MET consists of the reverse process in which cells gain apical polarity. These changes go forth to modify membrane filaments and reorganize the cell cytoskeleton in their process of differentiating from one tissual type to another. Certain molecular markers could be linked with these aforementioned processes, but these changes should rather be assessed by characteristic cellular modifications [66].

### 4.2. Direct Reprogramming—General Strategies for Different Tissues

New findings show that several binding proteins (i.e, Lin28 and Lin28b) have the capacity to block the process of maturing miRNAs. Therefore, miRNA metabolism is an important regulatory factor in reprogramming. Furthermore, several miRNAs in distinct combinations also possess the ability to induce reprogramming by themselves. Although very appealing, implementing this practice clinically has several inconveniences. Firstly, from a financial standpoint this would be difficult to obtain. Secondly, rejection of autologous grafts is not unheard of due to the immune mechanisms that are involved in the process [68]. These findings point to miRNA as a potential target in future cell replacement therapy research. A new role of apoptotic regulators in ESC (epithelial stem cells) modulation has arisen. Pro-apoptotic factors, such as p53, have been found to have an effect upon ESC differentiation. Caspase activation has shown to be of vital importance in reprogramming. Furthermore, inhibition of proapoptotic factors along with miRNA induction has been found to further ESC self-generation [69].

#### 4.2.1. Reprogramming Liver Cells into Insulin-Producing Cells (IPCs)

Due to the regenerative qualities of hepatic cells and the common embryological origin of both the liver and the pancreas during the early stages of organogenesis, liver cells have been a viable option for TD into pancreatic IPCs. Their common properties regarding insulin uptake and regulation and several transcription factors comprise a further argument in favor of exploring hepatic cells.

The basis of liver to pancreatic endocrine cell differentiation consists of PDX-1, an important transcription factor that has been shown to induce β-cell differentiation in other tissues. Thus, by using ectopic PDX-1, the activation of new β cells has been observed. In STZ mice, findings have proved to be significant as experiments have led to an improvement in insulin production.

Several additional pancreatic transcription factors (i.e., NEUROD-1, MAFA and NGN3) were similarly observed through their administration via multi-cistronic vectors. They were concluded to have a positive effect upon efficiency. Other factors used in order to improve liver to β-cell TD consisted of molecules such as β-cellulin, EGF, activin-4, exendin-4 and nicotinamide. These, along with chromatin agents that would be active in the process of histone modification have also been studied in order to properly assess their potential function in being added to reprogramming protocols. However, further analysis upon the immune response towards this type of therapy is needed, as T1DM consists of several autoimmune dysregulations and could lead to further impairment of transplanted cells [70,71].

Hepatic and pancreatic cell plasticity are of significant aid in the TD process. A further argument for the usage of liver cells is the presence of their mesenchymal qualities. Their origins are yet unknown, yet the hypothesis that these cells have undergone an EMT process has been put forth.

It has been observed that insulin production has been initiated in cells presenting as albumin positive. This could allow easier identification of cells that could be initiated into β cells. A better identification of such susceptible cells would be able to deliver a more targeted therapy without potentially damaging innate tissular functions [71,72]. Moreover, the trans-differentiation capacity to endocrine pancreas is not influenced by age, gender or metabolic state heterogeneity among different liver donors [73].

#### 4.2.2. Reprogramming Exocrine Pancreatic Cells into IPCs

Strong evidence from animal studies has shown that ductal tissue has the possibility of transdifferentiating into IPCs. This has been speculated upon due to the common embryologic origin of both exocrine and endocrine pancreatic tissues, therefore proposing them as a better source than hepatic cells. TD was shown through delivery of three key transcription factors that are known to have a significant importance during embryogenesis. Delivery of these factors in vivo (i.e., PDX1, Ngn3 and Mafa) has been attempted both through separate adenoviral vectors and through a single one. The latter was concluded to be of greater efficiency. However, a significant issue that arises is that the newly formed β cells will not aggregate as they physiologically should in islets, and this impediment undoubtedly impairs their functionality [74,75].

miRNAs’ involvement in embryogenesis and their potential in TD is detailed in Table 2. 

## 5. The Association of Specific miRNA with Diabetes Related Complications

Several miRNAs have been studied in relation to their possible role post β-cell transplant, but only miRNA-375 was deemed to have a significant statistical increased value. Any other miRNA tested so far have been found to have inferior utility. However, this raises research opportunities for observing miRNA-375 in association with several other possible biomarkers with the aim of increasing specificity in the early detection of damaged islet grafts [9]. Furthermore, several members of the miR-200 family have been noticed to have an antiapoptotic effect by targeting several networks, among which DNA (deoxyribonucleic acid) repair is included. Along with its presence in pediatric T1DM patients, it has also been correlated with other diseases [19].

The sizable rate of mortality and morbidity associated with a diabetes mellitus diagnosis is correlated with the complications it causes, specifically upon the neural and cardiovascular systems. The main cause of these is persistent high blood glucose values, which induce modifications, down to the cellular level [64]. Correlations have been found between extravesicular vesicles and accelerated healing of diabetic wounds. Processes of epithelialization and angiogenesis have been noticed in diabetic mice [64].

Several ongoing clinical trials are monitoring the effect of miRNAs and their therapeutic potential in monitoring disease evolution. One of the novel treatments for complications that is currently being observed is the usage of MSC (mesenchymal stem cell)-derived EVs. In addition to their potential role in transplantation, as mentioned in Section 3, they are being studied in relation to chronic kidney disease induced by diabetes. After administration, the eGFR (“*estimated glomerular filtration rate*”) was shown to have improved. This favorable effect could be due to the anti-inflammatory factors and cytokines brought by the MSCs, but further research is needed for a better understanding of their utility [64].

As diabetic retinopathy (DR) is a complication of drastic consequence (i.e., a leading cause of blindless in adults), studies have focused on better treatment plans. Several studies show miRs as specific towards patients with DM as opposed to their healthy counterparts. miR-126, miR-21, miR-181c, miR-1179, miR-21 and miR-155, miR-376a-1, miR-132, miR-125b, miR-100, miR-221, miR-200b, miR-146a, miR29b and miR-195 have been mentioned. However, a larger sample size of patients is needed for confirmation of previous findings. miR-21, -320a and -320b have also been found in the postmortem in the vitreous humor, showing their high specificity and sensibility for retinal disease [80,81]. Let-7a-5p could be correlated with increased proliferation of retinal cells, while miR-126 has been found to have lower values in DR. The miRNAs with most clinical value at this moment are miR-320a and miR-27b, which act through their interactions with thrombosponin-1 and subsequently VEGF (“*vascular endothelial growth factor*”) [80].

miR-195 has been found to be increased in several conditions of which we mention metabolic syndrome and glucose intolerance, as these are the most relevant to our review. After in vivo observations in STZ type 1 rat models, miR-195 is also known to be involved in regulating SIRT1 (sirtulin 1). The changes that occur in diabetic retinopathy are due to the oxidative stress upon SIRT1. These findings propose miR-195 as a possible therapeutic measure. Due to the complexity of the multifactorial results from diabetic retinopathy this will probably only be accomplished after combinational therapy [82].

miR-27b and miR-320a have been linked with diabetic retinopathy due to the fact of their effect on angiogenetic factors. Both have been shown to have an effect of TSP-1 which in turn inhibits VEGFc, leading to pathologic processes in retinal blood vessels. miRNA monitorization could be useful not only in initial diagnosis, but also within progressive retinopathy. However, the study did not take into account personal biological values, and the aforementioned miRNAs were not retina-specific. Therefore, further research is needed in order to correctly assess their applicability [83].

Furthermore, miR-133 and miR-192 have a role in diabetic heart (the former) and kidney (the latter) complications, while mi-R200b-3p has been shown to have an effect upon renal cell hyperplasia. This could later lead to diabetic nephropathy [22].

Diabetic nephropathy represents another common and difficult to manage chronic complication that DM patients present. In order to obtain a better understanding of miRs’ roles, urinary miRNAs have been analyzed. Several have been observed, but none were specific to kidney disease, suggesting their plasmatic origin. While miR-429 has been correlated with proteinuria and, therefore, kidney malfunction, miR-323b-5p was found to be dysregulated in long withstanding T1DM [84]. A metanalysis of 14 clinical studies found two miRs had significant value in regard to kidney disease. miR-126 and miR-770 have been found to have upregulated serum values in diabetic patients, while also presenting downregulation in urinary values. This could be suggestive of concomitant impairment of renal function [85]. Another promising finding is represented by the downregulation of miR-59-3p in patients presenting with diabetic nephropathy or T2DM patients. An increased level could, therefore, have a protective role upon the inflammatory factors at work. Its effect is speculated to be achieved through CX3CL1 tuning [86].

Further studies have brought forth miR-196 and miR-21 as correlated with renal fibrosis found in late-stage kidney disease. Conversely, the miR-200 and miR-29 families have an anti-fibrotic role as their downregulation in diabetic rodents has shown to favor the dysregulation of renal function. These findings could be used to map out new therapeutic targets. However, no miRNAs have been shown to be kidney specific, which brings about several difficulties. Instead, microbubble technology has been used in order to downregulate miR-21 in db/db mice. This has resulted in improved kidney function [87,88,89].

Several miRNAs have been also linked to diabetic neuropathy (DN) in diabetic rodents. Upregulated miR-199a-3p has been found to express accelerated disease progression, while miR-146 has been found in inflammatory cells present in peripheral neuropathy. miR499a has also been correlated with neuropathy. miR-155 has been found to have a protective effect in animal studies, by improving insulin sensitivity. The difficulty of further implementing these observations consists of the poor understanding of the time periods in which detection could suggest early subclinical neuropathy [90,91]. Naturally, recent research has shown potential biomarkers for DM that act through their role in mediating inflammation through their axonal regenerative properties. Furthermore, miRNAs have been found to be important factors in Schwann cell myelinogenesis and proliferation. These glial cells have been known to be of great importance when it comes to supporting axon regeneration [92].

However, most studies conducted on miRNAs’ role in DN have been largely preclinical, with few patients of both type 1 and type 2 enrolled in trials. However, genetic studies conducted on several polymorphisms of miR146, 128 and 21a genes have concluded that these modifications might have an important role to play within the inflammatory and metabolic pathways that undergo in DN progression. Extensive research must be continued for a better understanding [88,89,91].

miR-27b, along with miR-27a, has been found to be decreased in mice presenting with a dysfunctional epithelial layer. Due to the immune dysregulation that takes place in DM, wound healing will not be as effective as in healthy counterparts. This ultimately leads to one of the most dramatic complications of DM; diabetic foot ulcers [93]. Studies have been conducted in order to observe miRNAs as key factors in inflammation and healing processes. Several miRNAs have been detected in diabetics and could be useful in predicting foot ulcers. miR21 has been found to be decreased in the skin of people diagnosed with DM, highlighting it as a useful and specific target [93].

miRNA therapy through gene manipulation could have a beneficial impact. For example, removal of miR-155 in mice has been correlated with improvement in epithelial repayment. Although not associated directly with DM pathogenesis, miR-155 has an important part in mediating inflammation [93].

miR-146a has also been considered to play a role in inflammatory response. However, its upregulation has been correlated with autoimmune diseases in vivo in transgenic mice. Furthermore, treatment with MSCs has been shown to decrease inflammatory cells and improve wound healing, further showing miR-146a as an important mediator [68,93].

Upon revolutionary findings that ES-derived β cells can reverse DM in STZ mice, research has focused on finding efficient ways to replicate this phenomenon in humans [94]. While much progress has been made in the strenuous processes of differentiating β cells from pluripotent stem cells, much remains to be resolved. The steps which the ideal protocol should take would be able to differentiate mesenchymal cells all the way to fully-functioning, insulin secreting β-cells. However, despite the difficulty that reproduction of any such protocols brings about, the obtained cells are low in number, hardly enough to ensure sufficient insulin secretion [95]. Further impediments that have been encountered consist of the difficulty in maturation of endocrine pancreatic progenitors. Because of this, several new strategies have been looked into. Among these, miRNAs and their diverse roles in islet maturation and insulin production could be of great further use, though many valuable details remain unknown. Epigenetic factors and their effects also are a new potential target considering β-cell maturation, along with nitric oxide (NO). The latter has been shown to have important roles regarding gene expression. A better understanding of these factors might bring about further progress [95]. Most recent achievements in this field consist of skipping the late in vitro stages of differentiation. Instead, the cells were placed directly in vivo, transfected into STZ mice in order to see whether the differentiation process could continue. Unfortunately, no significant changes occurred under observation [95]. New research has proposed a seven-step protocol that aims to better the process by monitoring several transcription factors. Among these, the co-expressed PDX1 and NKX6.1 factors are taken into account for pancreatic progenitor cells, followed by NGN3, NEUROD1, NKX2.2 and finally MAFA. The aforementioned protocol has been found to reserve DM after cell transplantation in mice [96].

## 6. Bioinformatics Resources for miRNA Analysis Moving towards Precision Medicine

As the importance of miRNAs in the modulation of other multiple pathogenic pathways has become clearer, more approaches are being undertaken to develop miRNAs as candidate biomarkers. Therefore, research has breached beyond searching for the many roles that miRNAs and other noncoding RNA have, and towards implementing them as mimics or repression in order to modulate their transcription. Therefore, development in this area of study will arise [97]. Various miRNAs have been studied extensively, due to their important role in DM pathogenesis, inflammation, disease, β-cell destruction and many other diseases, such as malignant tumors and autoimmune dysfunctions. While many have been found to have clinical potential, so far few have reached incipient stages of clinical trials. Anti-miRNA-103 and anti-miRNA-107 have managed to approach phase I trials, targeting insulin resistance in T2DM patients [98]. A recent populational case-control study of 90 participants, both healthy, diabetic and prediabetic, highlighted an increase in miR-122, concomitant with the rise in inflammatory cytokines, such as IL-6 and TNF-α, in both prediabetic and diabetic patients, in comparison to the control group. This rise was also correlated with high HOMA-IR (homeostasis model assessment-estimated insulin resistance) concentrations. Conversely, miR-126-3p and miR-146a were found to be decreased in patients exhibiting various degrees of insulin resistance. However, as this study had a small sample size of participants, it was of lower statistical significance [99]. Despite the clear utility of miRNAs that we have detailed so far, identifying target genes is still full of impediments and technical difficulties. Several bioinformatic tools have been developed and updated over the years, but as there are still many unknown factors regarding miR-mRNA interactions, these methods have often produced a large number of false positive results [100].

The existent web platforms are used for determining the statistical significance of several findings in regard to miRNA target interactions. Moreover, the issue of conflicting databases and differences among storage methods have also proved to be a significant impediment. MIENTURNET offers a centralized database of several existing web-based applications (see Table 3) [100]. The transcriptional mechanism that miRNA is part of is the basis of these online databases. Most search for a suitable match between miRNA and mRNA. In animal organisms, a perfect matching is rare. Instead, the seed region of a certain miR is used to search for its potential match. It is searched to be complementary with the 3′UTR (“untranslated region”) of the mRNA that it ends up interacting with [68]. Both TargetScan and miRTarBase perform analysis upon several miRNA targets. The results are filtered based the strength of existing experimental evidence. MIENTURNET also facilitates in identifying the target genes, depending on the p-value, which shows the level of statistical significance of findings. Thus, in regard to insulin secretion, after functional enrichment analysis has-miR-128-5p has been found to be most significant, followed by has-miR-9-5p and has-miR-29-3p [100].

## 7. miRNAs as Biomarkers of Insulin Resistance: Using miRNAs Delivery in Ameliorating Metabolic Disorders in Preclinical Trials

Insulin resistance (IR) depends on several factors and is strongly correlated with T2DM. Its pathogenesis relies on several factors and biochemical substrates, each of the subsequent processes being strongly interconnected with miRNAs [6]. Among several of its other roles and possible utilities that have been detailed so far, miRNA also acts as a marker of insulin resistance in several peripheral tissues. Thus, several miRNAs have been correlated with T2DM and metabolic syndrome due to their muscular, hepatic and adipose tissue expressions. Furthermore, they have also been correlated with lipoprotein and cholesterol metabolism [114]. Several miRNAs have been looked into due to their potential as biomarkers in T2DM as they could have an important role in monitoring disease progression and treatment. Several miRNAs have been found, some more statistically significant than others due to the differences in their individual parameters from one study to another. A metanalysis of the literature has taken this subject under evaluation. It was concluded that miR-103, miR-107, miR-132, miR-144, miR-142-3p, miR-29a, miR-34a and miR-375 have significant modifications in peripheral blood levels. Similarly, miR-199a-3p and miR-223 were found to have dysregulated tissular values in T2DM. The aforementioned miRNAs had statistical significance which marks them as potential biomarkers in insulin resistant DM [115]. Among the targeted miRNAs, a few have been found to have dysregulated expressions in T2DM patients. The miRNAs in question consist of miR-126, miR-21, miR-27a, miR-27b and miR-130a. These finding might show more information regarding the organism’s response to high blood sugar values [68].

The correlation between miR-126 lowered expression with a higher risk of T2DM development in a short span of time should be further analyzed [6].

The importance of the interconnection of obesity, metabolic syndrome, inflammation and T2DM manifestations must also be considered. As DM and obesity themselves are permanent sources of systemic inflammation and ROS (“Reactive Oxygen Species”) production, miRNAs associated with increased ROS have been suspected of having important regulatory roles [98].

miR-33 has been shown to increase obesity levels in mice, even those not on high-fat diets eventually manifesting IR, which proves the multifactorial nature of T2DM. miR-335 was found to be correlated with inflammation in obesity, through TNF-α modulation. Dysregulated miRNAs in obesity and metabolic syndrome are also correlated with low adiponectin levels [116].

miR-18a and miR-34c are suspected as possible biomarkers for metabolic syndrome as they were found to be decreased in patients exhibiting this condition, compared to the control group. Furthermore, their decreased levels were inversely correlated with the high levels of Il-6 and cortisol. However, the study that overviewed these observations was not composed of a significantly large sample size of participants [116].

miRNAs have also been found to be of vital importance in adipogenesis, an important factor that leads to T2DM and IR. Several miRNAs, among which we mention miR-103, -143, -221 and -222, have been correlated in animal studies on mice, proving to have an important role in the biochemical pathways that lead to IR. These aforementioned pathways consist of upregulation of certain transcription factors and factors that have a vital role in lipid and glucose metabolism (Fabp4, respectively, Glut4). Furthermore, miR-103 is correlated with adiponectin, a key component in developing obesity and metabolic syndrome. These changes are also correlated with the concomitant inflammation and upregulated TNF-α expression [117].

Furthermore, miR-132 and miR-124a have been found to be cerebral and pancreatic regulators of glucose metabolism. Additionally, miR-30a-5p and miR-195 have been found to target BDNF; an important element in the dysregulation of appetite, studied in regard to several genetic diseases such as Wilms’ tumor [117]. Another important element that leads to IR is hepatic insulin dysfunction, concomitant with NAFLD (non-alcoholic fatty liver disease). Both long and short noncoding RNAs have been observed so as to determine their role and potential in the prognostic value of disease progression. miR-122-5p, miR499-5p and miR-802 have been mentioned in the literature [118]. EVs (“Extracellular Vesicles”) have also been correlated with a high rate of obesity and insulin resistance. Adiponectin has been found in circulating EVs, proving a relationship with insulin sensitivity. Furthermore, animal studies have also found that injection in VAT (“Visceral Adipose Tissue”) EVs increases inflammatory cytokines, such as IL-6 and TNF-α, a modified glucose tolerance. Improvement in IR in T2DM patients has been correlated with a normalization of let-7a and let-7f levels [16].

One of the conventionally used pharmaceutically active compounds used in the management of insulin resistance is metformin. Metformin has been found to also have effect upon various miRs which sets it apart as an effective therapeutical approach. Its effect upon miR-34a has shown to provide additional protection against neurodegeneration in DM. Thus, approaching therapy in regard to its regulation of miRNAs could be a step forward in clinical applicability [119,120].

An important limitation of using serum biomarkers in diseases that target specific tissues consists of the wide error range. We might therefore obtain inconsistent results that cannot be correlated with pathophysiological damage. We must also take into consideration the scarcity of such biomarkers, particularly in the time period which is of most interest, before any clinical signs and/or symptoms. Thus, the relevance of dosing such miRNA is lowered. Along with the heterogeneity of miRNA, these are several important obstacles one needs to overcome in order to be able to use them for effective T1DM management. This is why miRNAs that target Treg cells are specially noted due to their direct role in autoimmunity [2,7]. Moreover, the exosomes that have been generally used in such studies are mostly heterogenous populations. For an increased specificity, development of more markers should be researched. Therefore, identification of different EV subtypes could be accomplished [121].

We have pinpointed some discrepancy between studies regarding the roles of certain miRNAs, largely due to the varying degrees of patients that were taken into consideration, small groups generally being used. More standardized studies that look into the conflicting areas in literature regarding this topic would be of great use for further progress [4].

Another issue that arises is that despite the immune mechanisms implicated in the pathogenesis of T1DM, pancreatic anomalies also pose an importance. This only furthers the need to identify tissue-specific exosomes [121]. Several problems in regard to large-scale production and storage of EVs also need addressing [64].

Furthermore, the low number of researched miRNAs that have rendered significant results in clinical studies as they have yet to reach stage III trials may have significant side-effects. This is due to their interactions with several genes as multiple miRNAs could modulate the same mRNA and thus could regulate multiple pathological pathways.

The negative charge of the majority of miRNAs, in contrast to PNAs (“*Peptide Nucleic Acids*”) or siRNAs (“*small inhibitory RNA*”), also provides a higher degree of unwanted nonspecific tissular accumulation [122,123]. These biochemical interactions limit the precision of targeted gene therapy and could warrant side effects. In order to minimize such risks, future research should focus on narrowing the spectrum of miRNAs to those with the highest specificity for the disease in question. In addition, better control in delivery and stability of miRNA modulators would also greatly advance therapeutic potential. Several possible solutions are being investigated, such as using platelet-derived EVs or exosomes, which had been shown to have great potential. The latter have been found to bridge biological barriers and impose the least side effects [122,124].

Other options currently in research for miRNA gene targeting are gamma PNAs and plant-derived miRNAs as they both present lower toxicity levels. Thus, gamma PNAs have a shorter half-life and weaker bounds to serum proteins, and plant-derived exosomes have superior intestinal uptake [124].

## 8. Conclusions and Future Perspectives

As DM patient cases keep rising, the problem of early diagnosis and treatment is essential. While modern medicine has several diagnostic tools at its disposal, miRNAs could be a useful non-invasive biomarker to be followed, both for β-cell damage and insulin resistance. miRNAs represent a broad spectrum of utilities for both early detection of DM and efficient monitoring of disease progression. Further progress in this line of studies will potentially bring about superior treatment plans as we approach precision medicine. Regarding whether this treatment refers to stem-cell usage, trans-differentiation or through upregulating miRNA via genetic methods, further knowledge regarding the functions and utilities of miRNAs in DM could broaden the spectrum of possible treatment plans. Technical limitations regarding both laboratory processing and subject analysis must also be taken into account. More original studies with a larger number of DM patients are necessary to broaden the existent database of possible miRNAs that could be used as biomarkers and provide compelling evidence for their clinical implementation.

## Figures and Tables

**Table 2:** miRNAs involved in different organs’ TD process into pancreas.

miRNAs	Findings	Reference
miR-375	Overexpression of miR-375, combined with concomitant underexpression of miR-9 has been shown to aid in the TD of mesenchymal stem cells into IPCs. This was achieved without the use of other regulating factors.	[76]
miR-9		
miR-302	Human liver cells could be able to TD into IPCs via miR-302 induction, to which an array of maturation factors are also added. Among these we mention PDX1, Ngn3 and Mafa.	[77]
miR-181c-5p	miR-181c-5p was upregulated in the process of stem cell formation in pancreatic endocrine cells. This process undergoes through pathways that repress Smad7 and TGIF2. miR-181c-5p was found to be upregulated in β-cells while downregulated in hepatocytes which suggests a higher specificity for TD into functional β-cells.	[78]
miR-34c	Through its target genes, miR-34c was essential in the generation of IPCs from MSCs. Among the aforementioned targets we mention SARA1A, ACSL4, PDE7B, MAP2K1 and PDGFRA.	[79]
miR-294/302 let-7	While the miR-294/302 family was found to hinder mesenchymal processes, the let-7 family acted conversely by promoting such. Tgfbr2 was shown to be a target of miR-294/304; its regulation had an impact on MET and the efficiency of the reprogramming process.	[69]
miR-7	It was shown to have a vital role in pancreatic embryogenesis and differentiation of endocrine cells into α-and β-secreting cells. Its role was fulfilled through Pax6 regulation, an important transcription factor in generating optimal levels of pancreatic hormones.	[58]

**Table 3:** Algorithms and tools for identifying miRNAs and their target.

Resource	Link	Main Findings	References
MIENTURNET	http://userver.bio.uniroma1.it/apps/mienturnet Accessed 8 June 2022	Reunites several miRNA comprehensive databases, such as TargetScan and miRTarBase (see below) in order to provide a comprehensive analysis of miRNA target-interactions	[100]
Target Scan	https://www.targetscan.org/vert_80/ Accessed 8 June 2022	Predicts miRNA targets through algorithms that match seed regions to complementary sites	[101]
MirTargetLink2	https://ccb-compute.cs.uni-saarland.de/mirtargetlink2 Accessed 8 June 2022	Searches for miRNA targets from human, mouse and rat species. It displays miRNA interactions through visual networks	[102]
DIANA-microT	http://diana.imis.athena-innovation.gr/DianaTools/index.php?r=MicroT_CDS/index/ Accessed 8 June 2022	miRNA target prediction and analysis of miRNA–mRNA interactions	[103,104]
sRNAbench	https://arn.ugr.es/srnatoolbox/ Accessed 8 June 2022	Successor of the miRanalyser program prom 2009. This resource provides small RNA profiling	[105]
PicTar	https://pictar.mdc-berlin.de/ Accessed 8 June 2022	Detects interactions of miRs with their respective targets	[106]
PITA	https://genie.weizmann.ac.il/pubs/mir07/mir07_prediction.html Accessed 8 June 2022	Provides detection of potential miR targets via scanning of UTR sequences	[107]
miTEA	http://cbl-gorilla.cs.technion.ac.il/miTEA/ Accessed 8 June 2022	Provides enrichment analysis of GO (Gene Ontology), filtering the results by p-value	[108,109]
mirNET	https://www.mirnet.ca/ Accessed 8 June 2022	Analyzes miRNA interactions with target genes and their binding sites. Adds data regarding TFs (Transcription Factors) and SNPs (Single-Nucleotide Polymorphisms).	[110,111]
miRDB	http://mirdb.org/ Accessed 8 June 2022	Predicts miRNA interaction targets. Predicts processes that undergo miRNA regulation. Uses GO data.	[112]
RNAhybrid	https://bibiserv.cebitec.uni-bielefeld.de/rnahybrid Accessed 8 June 2022	Searches potential target sequences of statistical significance.	[113]

## Data Availability

Not applicable.
